# A dataset on multi-trait selection approach for the evaluation of F1 tomato hybrids along with their parents under hot and humid conditions in Bangladesh

**DOI:** 10.1016/j.dib.2024.110859

**Published:** 2024-08-22

**Authors:** Mohammad Matin Akand, Mohammad Mostafa Kamal, Md. Iqbal Haque, Shyamal Brahma, Mohammed Nure Yousuf, Mahmuda Khatun

**Affiliations:** a Bangladesh Agricultural Research Institute, Gazipur 1701, Bangladesh; b Department of Agricultural Extension, Mirzapur, Tangail 1940, Bangladesh

**Keywords:** Summer tomato, Selection gains, Heritability, Multicollinearity, MGIDI index, Strengths and weaknesses of hybrids

## Abstract

This dataset aims to evaluate the use of multiple trait-based selection methods with multi-trait genotype-ideotype distance index (MGIDI) models to identify superior summer F1 tomato hybrids suitable for the climatic conditions of countries like Bangladesh. The dataset was generated using 14 cross combinations from a Line × Tester mating design, along with seven parental lines and two tester parents of tomatoes with diverse genetic bases and heat tolerance qualities in a randomized complete block (RCB) design. The likelihood ratio (LR) test indicated highly significant genotype effects for most of the analyzed traits. A heatmap of correlation analyses between 16 traits identified a highly significant positive correlation (*r* > 0.8) between NFrPC and NFPC and between AFW and FW, preliminarily indicating a clear trace of multicollinearity among these traits. The traits NFPP, YPP, and Yield showed the highest predicted genetic gains, indicating their potential for substantial improvement through selection. Additionally, the heritability estimates ranged from 0.54 to 0.99, highlighting high heritability across the traits, which suggests favourable conditions for effective selection strategies. The strengths and weaknesses of hybrids AVTOV1002×C41 and AVTOV1010×C41 were evaluated based on their contributions to MGIDI across four major factors. These hybrids demonstrated strong performance, particularly excelling in traits associated with FA1, FA2, and FA4. The dataset of MGIDI can be universally applied to rank treatments based on desired values of multiple traits, with its potential for rapid expansion in evaluating various types of plant experiments.

Specifications Table

**Table:** 

Subject	Agricultural Science, Horticulture, Genetics, Data Mining and Statistical Analysis.
Specific subject area	Evaluation of F1 summer tomato hybrids and their parents under hot and humid conditions using the multi-trait genotype-ideotype distance index (MGIDI) statistical model.
Type of data	Raw, Analyzed, Table, Figure
Data collection	The seeds of the F1 hybrids and their parents were sown in a well-prepared seedbed. Forty-day-old tomato seedlings were then transplanted into the main field under transparent polytunnels. The polytunnels were 2.3 m wide and contained two-unit beds, each measuring 0.8 m by 1 m, with a 30-cm drain between the 14-unit beds. Each unit bed had double rows, accommodating 24 plants. Most of the data were collected from randomly selected plants—five plants per parental line and their crosses. Fruits per plant, yield per plant, and yield per hectare were calculated from the plot yield.
Data source location	The experiment was conducted at the vegetable experimental field under the polytunnels of the Horticulture Research Centre (HRC), Bangladesh Agricultural Research Institute (BARI), Gazipur-1701, Bangladesh (23°59′27.7″N 90°24′42.4″E, 8.4 masl).
Data accessibility	Repository name: Mendeley Data identification number: 10.17632/k78cc8s7hg.1 Direct URL to data: https://data.mendeley.com/datasets/k78cc8s7hg/1
Related research article	none

## 1 Value of the Data

• The dataset of the multi-trait genotype-ideotype distance index (MGIDI) helps select superior treatments/genotypes in plant experiments by combining desired traits, enhancing breeding efficiency, and reducing reliance on univariate analyses. Its straightforward, graphical approach allows quick interpretation and application of results, identifying effective traits and balancing strengths and weaknesses.
• Farmers and agricultural practitioners can optimize resource allocation by choosing the best-performing hybrids for cultivation, leading to improved resource utilization and increased productivity.
• Other researchers can reuse these datasets to validate and further develop the MGIDI index in different agricultural contexts or crop species, expanding its applicability and refinement.

## 2 Background

Tomato (Solanum lycopersicum L.), of the Solanaceae family, is widely grown in Bangladesh and other parts of the world for its taste, nutritional value, uses, and commercial importance [[1], [2], [3], [4], [5]]. In developed countries, hybrid tomatoes are popular for their high yield and quality. Still, in Bangladesh, hybrid seed use is limited, necessitating the development of high-yielding, high-quality, and widely adaptable hybrid varieties. In Horticultural experiments, evaluating multiple traits is common, but identifying genotypes/treatments that excel across many traits is challenging. Researchers often choose univariate analyses and post-hoc tests for mean comparisons, suggesting that multi-trait framework benefits may be underutilized. Classical linear multi-trait selection indexes exist, but multicollinearity and arbitrary weighting coefficients can hinder genetic gains [[6], [7], [8]]. In this dataset, we have used the MGIDI (Multi-trait Genotype-Ideotype Distance Index), introduced by Olivoto and Nardino [7], which offers a novel approach to selecting genotypes and recommending treatments based on multiple traits. MGIDI provides more efficient and accurate treatment recommendations by focusing on desired or undesired crop characteristics. It is unique, easy to interpret, and free from weighting coefficients and multicollinearity limitations.

## 3 Data Description

### 3.1 Variance components, genetic parameters and phenotypic correlations

**Fig. 1:**
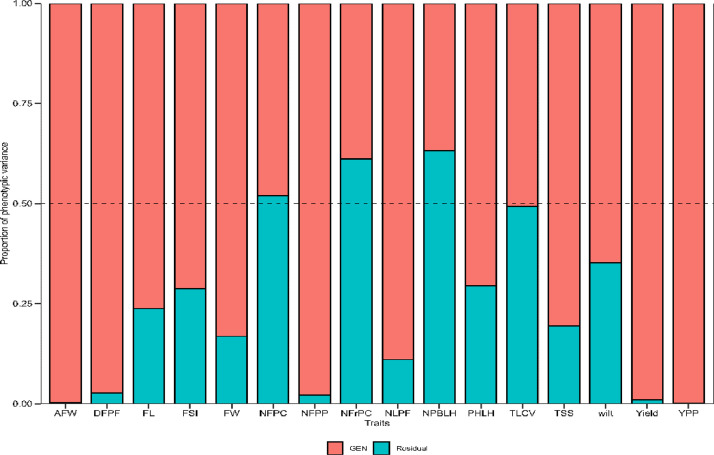
Estimated variance components for the traits evaluated. See Table 4 for the full trait names.

**Fig. 2:**
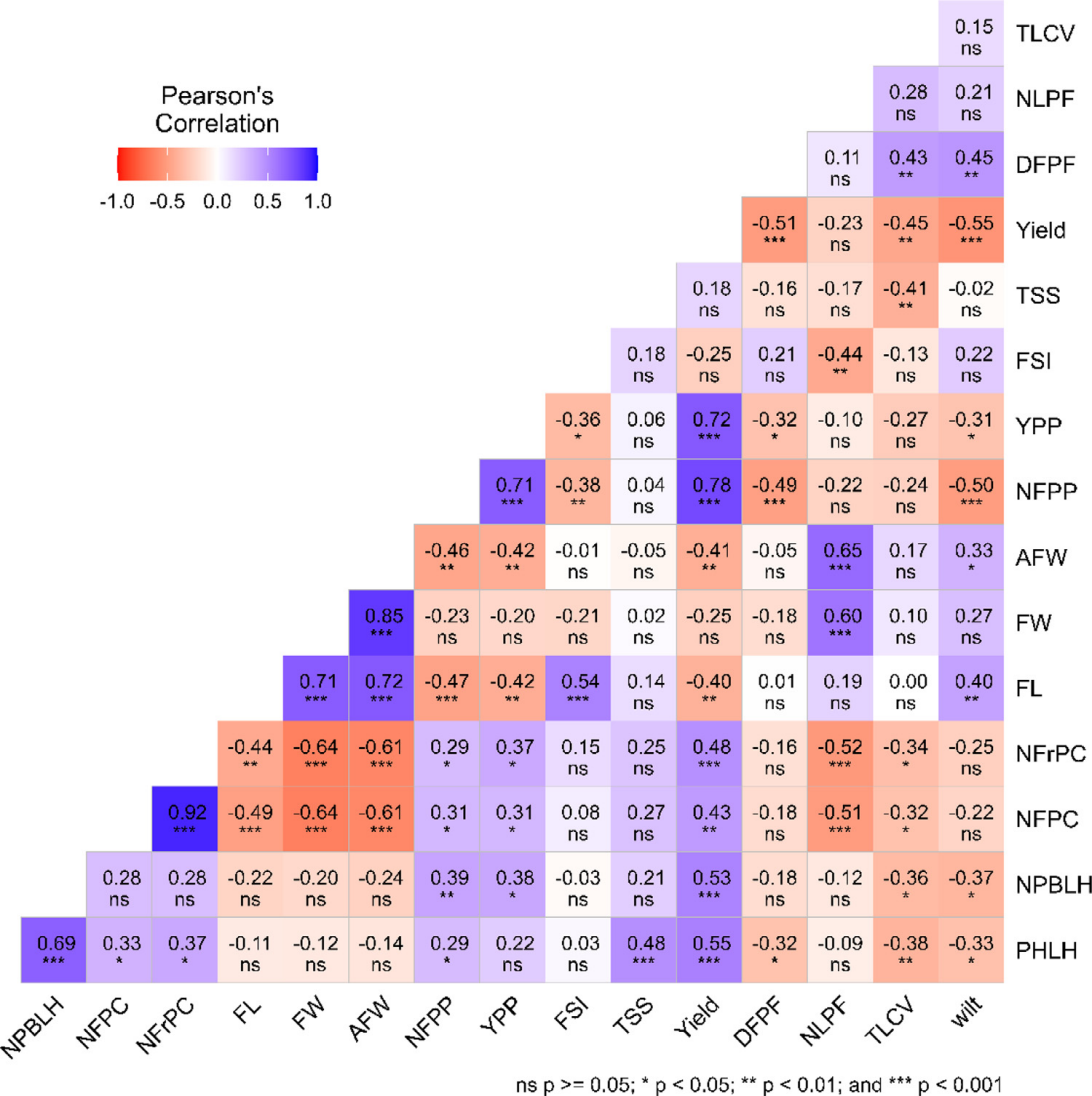
Phenotypic correlation heatmap between the traits evaluated (The practice of genotype selection involved a preliminary examination of traits that contribute to multicollinearity). See Table 4 for the full trait names.

The likelihood ratio (LR) test indicated highly significant genotype effects (*p* < 0.01) for most of the analyzed traits. Except for NPBLH, NFPC and NFrPC, all the other traits had the genotypic variance (σ^2^g) as the main component of the phenotypic variance (σ^2^p) (Tabel 1; Fig. 1). The broad-sense heritability on a genotype mean basis (h^2^) ranged from 0.37 (NPBLH) to 0.99 (AFW). High values of heritability (h^2^ > 0.8) were observed for FW, AFW, NFPP, YPP, Yield, DFPF and NLPF, suggesting good prospects of selection gains for these traits. The assessment of accuracy (AS) for the mean trait value showed significant genetic variation among the genotypes, with an accuracy level greater than 0.70, enabling precise prediction of the genetic value of the trait. A heatmap of correlation analyses was conducted between 16 traits to preliminarily identify those contributing to multicollinearity (Fig. 2). A highly significant positive correlation (*r* > 0.8) was found between NFrPC and NFPC, as well as between AFW and FW.

**Table 1:** Deviance analysis and genetic parameters for traits evaluated.

Traits	*LTR*	*AIC*	Genetic parameters						
			*σ^2^g*	*σ^2^p*	*h^2^*	*AS*	*CVg*	*CVr*	*CV ratio*
PHLH	15.17***	430.23	551.59	781.39	0.71	0.91	14.47	9.34	1.55
NPBLH	3.22ns	199.28	1.51	4.11	0.37	0.73	13.49	17.65	0.76
NFPC	5.77**	125.37	0.37	0.77	0.48	0.81	16.62	17.29	0.96
NFrPC	3.60ns	109.16	0.21	0.53	0.39	0.75	19.18	24.06	0.80
FL	19.15***	114.72	0.46	0.60	0.76	0.93	14.06	7.85	1.79
FW	25.91***	94.69	0.32	0.38	0.83	0.95	12.36	5.56	2.22
AFW	115.68***	442.04	1019.36	1022.02	0.99	0.99	45.41	2.32	19.56
NFPP	68.80***	360.35	156.10	159.64	0.98	0.99	36.53	5.50	6.64
YPP	132.92***	92.25	0.36	0.36	0.99	0.98	32.71	1.13	28.98
FSI	15.62***	−47.67	0.01	0.01	0.71	0.91	9.75	6.19	1.58
TSS	23.10***	106.11	0.40	0.49	0.81	0.94	11.76	5.76	2.04
Yield	86.94***	331.22	81.55	82.35	0.99	0.99	25.71	2.54	10.13
DFPF	64.42***	285.63	28.43	29.22	0.97	0.99	16.19	2.70	5.99
NLPF	34.45***	138.28	0.91	1.03	0.89	0.97	28.13	9.92	2.84
TLCV	6.55**	275.86	11.87	23.40	0.51	0.82	16.81	16.56	1.02
wilt	11.95***	305.77	29.89	46.18	0.65	0.89	33.51	24.73	1.36

**Notes:** ****, ** and * significant at <0.0001, <0.01 and <0.05 respectively; ns – not significant. *LRT*, Likelihood ratio tests for genotype; *AIC*, Akaike's Information Criterion for the selected model; *σ^2^p*, phenotypic variance; *h^2^*^,^ heritability; *As*, the accuracy of genotype selection; *CV_g_* and *CV_r_*, the genotypic and the residual coefficient of variation, respectively; *CV ratio*, the ratio between genotypic and residual coefficient of variation. See Table 4 for the full trait names.

### 3.2 Factor analysis and predicted selection gains

Four principal components were retained, which explained 79.6 % of the total variation among the traits (Table 2). Thus, it was possible to reduce the data dimensionality by 75 % keeping a high explanatory power. After varimax rotation, the average communality (*h*) was 0.796 (wilt 0.52 ≤ *h* ≤ 0.94 FW), indicating that a high proportion of each variable's variance was explained by the factors. The 16 traits were grouped into the four factors (FA) as follows: In FA1 the fruit-related traits FL, FW, and AFW with positive loadings, and NFPC, NFrPC and NLPF with negative loadings; In FA2 the traits NFPP, YPP, Yield, DFPF and wilt (with positive loadings); In FA3 the traits FSI, TSS and TLCV (with positive loadings) and in FA4 the plant-related traits PHLH and NPBLH (with negative loadings) (Table 2).

**Table 2:** Eigenvalues, explained variance, factorial loadings after varimax rotation, and communalities obtained in the factor analysis.

Variables	FA^1^	FA^2^	FA^3^	FA^4^	Communality (*h*)	Uniquenesses
PHLH	−0.05	0.25	0.17	**−0.87**	0.86	0.14
NPBLH	−0.23	0.26	−0.11	**−0.84**	0.83	0.17
NFPC	**−0.82**	0.23	0.18	−0.23	0.81	0.19
NFrPC	**−0.82**	0.25	0.23	−0.31	0.89	0.11
FL	**0.70**	−0.28	0.57	0.10	0.90	0.1
FW	**0.97**	0.05	0.03	0.05	0.94	0.06
AFW	**0.92**	−0.18	0.11	0.06	0.90	0.1
NFPP	−0.28	**0.79**	−0.24	−0.19	0.79	0.21
YPP	−0.27	**0.63**	−0.27	−0.28	0.62	0.38
FSI	−0.19	−0.45	**0.76**	0.03	0.81	0.19
TSS	−0.02	0.02	**0.56**	−0.54	0.60	0.4
Yield	−0.28	**0.75**	−0.04	−0.49	0.88	0.12
DFPF	0.16	**0.85**	0.3	−0.02	0.85	0.15
NLPF	**−0.75**	0.12	0.51	0.09	0.85	0.15
TLCV	−0.16	0.45	**0.58**	−0.36	0.69	0.31
wilt	−0.22	**0.67**	−0.06	−0.14	0.52	0.48
Eigenvalues	6.5	2.81	2.33	1.1		
Variance (%)	40.6	17.6	14.6	6.89		
Accumulated (%)	40.6	58.2	72.7	79.6		
communalities' mean	0.796					

**Note:** the superscript numbers 1, 2, 3, and 4 represent FA1, FA2, FA3 and FA4, respectively, where bold values indicate the variables grouped within each factor. See Table 4 for the full trait names.

The predicted genetic gain (SG) for effective traits in the MGIDI index is presented in Table 3. Results indicated a higher SD% for major measured traits, such as NFPP, YPP, Yield, AFW and TSS. The estimates of heritability on the entry-mean basis ranged from 0.54 (NPBLH) to 0.99 (AFW, NFPP, YPP Yield and DFPF), which were high for all filtered traits. This suggests that there are good prospects of selection gains for these traits. The selected traits with the highest genetic gains (SG%) were NFPP (32.70 %), YPP (29.90 %), and Yield (21.90 %). The only trait with undesired selection gain (–21.50 %) was AFW.

**Table 3:** Predicted genetic gain for the effective traits in the MGIDI index.

Trait	Factor	X_o_	X_s_	SD	SD (%)	h^2^	SG	SG (%)	sense	goal
NFPC	FA1	3.65	3.75	0.10	2.70	0.65	0.06	1.75	increase	100
NFrPC	FA1	2.37	2.51	0.15	6.15	0.56	0.08	3.44	increase	100
FL	FA1	4.81	4.92	0.11	2.21	0.87	0.09	1.91	increase	100
FW	FA1	4.55	4.58	0.03	0.67	0.91	0.03	0.60	increase	100
AFW	FA1	70.30	55.20	−15.10	−21.50	0.99	−15.10	−21.50	increase	0
NLPF	FA1	3.40	3.02	−0.37	−11.00	0.94	−0.35	−10.40	decrease	100
NFPP	FA2	34.20	45.50	11.30	33.00	0.99	11.20	32.70	increase	100
YPP	FA2	1.83	2.38	0.55	29.90	0.99	0.55	29.90	increase	100
Yield	FA2	35.10	42.80	7.72	22.00	0.99	7.68	21.90	increase	100
DFPF	FA2	32.90	30.00	−2.89	−8.79	0.99	−2.85	−8.67	decrease	100
wilt	FA2	16.30	15.10	−1.22	−7.49	0.79	−0.96	−5.89	decrease	100
FSI	FA3	1.06	1.07	0.01	1.26	0.83	0.01	1.05	increase	100
TSS	FA3	5.37	6.09	0.72	13.40	0.89	0.65	12.00	increase	100
TLCV	FA3	20.50	16.80	−3.75	−18.30	0.67	−2.52	−12.30	decrease	100
PHLH	FA4	162.00	172.00	9.42	5.81	0.83	7.80	4.81	increase	100
NPBLH	FA4	9.12	9.97	0.85	9.33	0.54	0.46	5.03	increase	100
Total (Increase)								115.09		
Total (Decrease)								−58.76		

**Notes:** X_0_ = overall mean, X_S_ = mean of selected hybrids and their patents, SD = selection differential, h^2^ = broad-sense heritability on the entry-mean basis, SG = selection gain, goal = selection gains match desired sense (100 for yes and 0 for no). See Table 4 for the full trait names.

### 3.3 Treatment ranking according to the multi-trait index

**Fig. 3:**
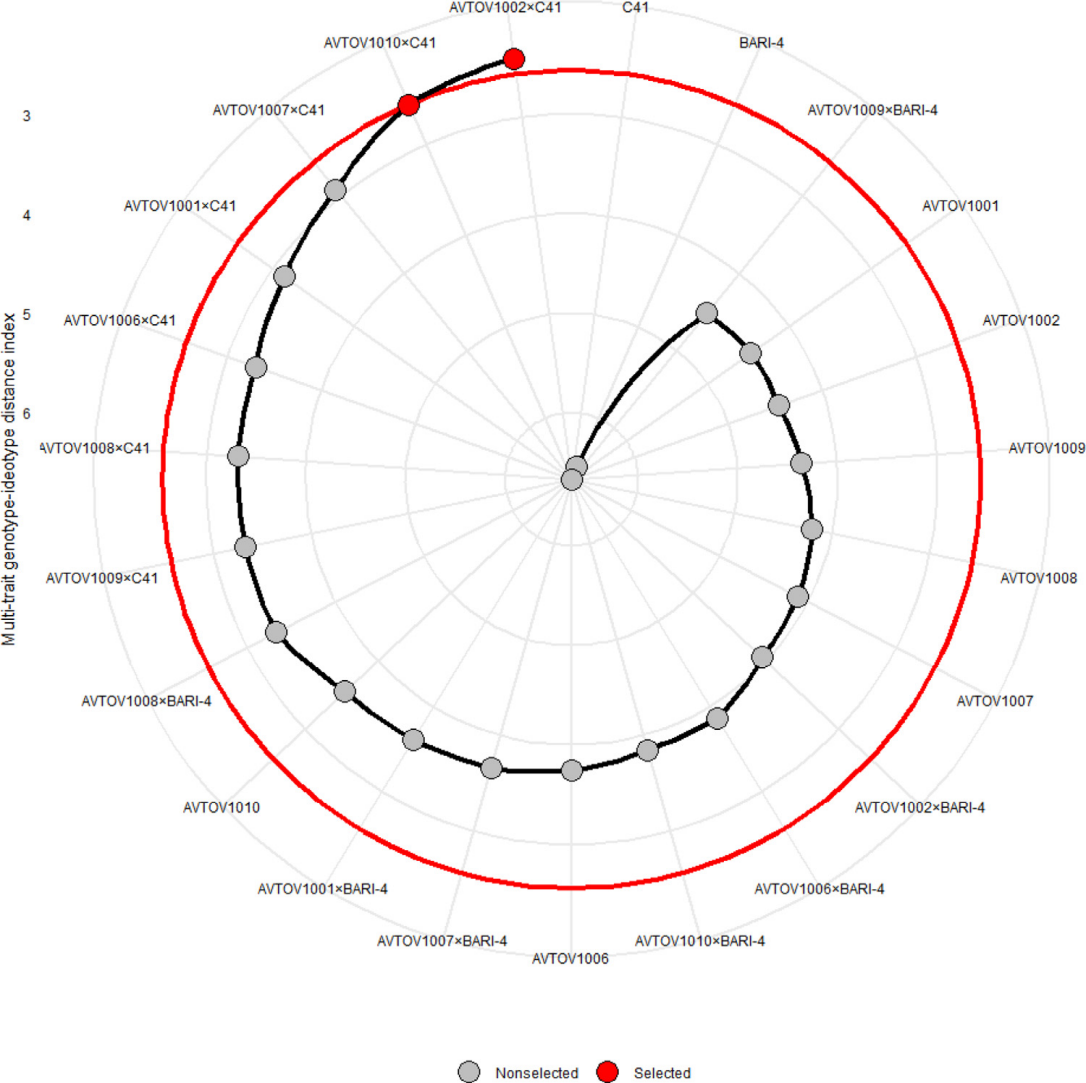
Treatment ranking based on the MGIDI index (The selected genotypes are shown in red and the unselected in black circles in the electronic version of the article. The circle represents the cut point according to the selection pressure ≈10).

Fig. 3 presents a brief visual illustration of the rankings of genotypes according to their MGIDI index values, and highlights selected genotypes based on the given selection criteria. Out of all the genotypes, AVTOV1002×C41 and AVTOV1010×C41 were selected and highlighted in red, indicating their significant performances. Additionally, two other genotypes, AVTOV1007×C41 and AVTOV1001×C41, were also ranked among the top four best genotypes based on their performance across multiple traits. These genotypes possess favourable characteristics for the given traits, making them suitable for the study or the desired purpose.

### 3.4 Strengths and weakness of hybrids

**Fig. 4:**
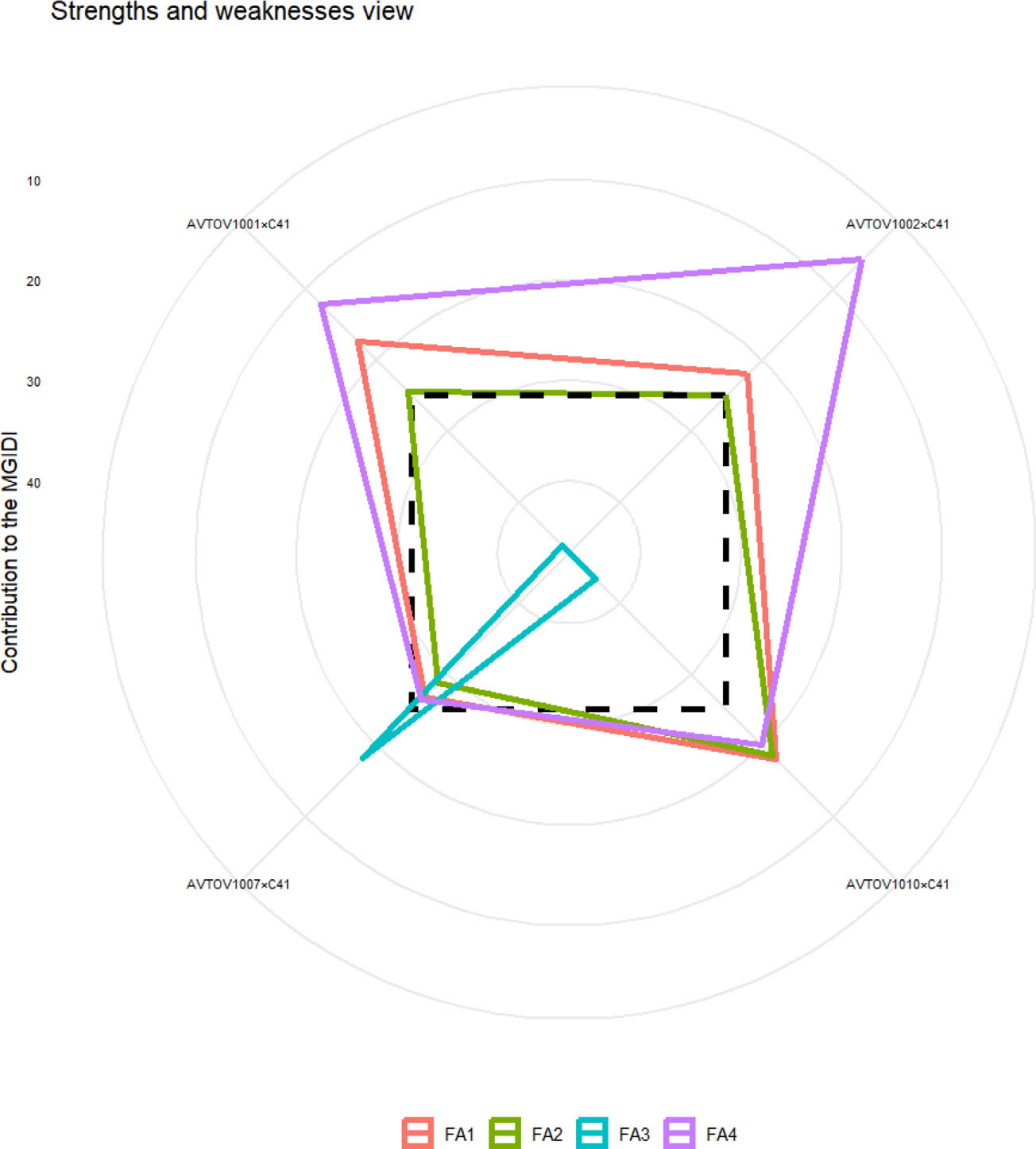
The strengths and weaknesses view of the selected genotypes is shown as the proportion of each factor on the computed multi-trait genotype–ideotype distance index (MGIDI). The smaller the proportion explained by a factor (closer to the external edge), the closer the traits within that factor are to the ideotype. The dashed line shows the theoretical value if all the factors had contributed equally. The traits grouped into each factor where: FA1: NFPC, NFrPC, FL, FW, AFW and NLPF; FA2: NFPP, YPP, Yield, DFPF and wilt; FA3: FSI, TSS and TLCV and FA4: PHLH and NPBLH.

Fig. 4 represents the strengths and weaknesses of the genotype, labelling the contribution of factors toward MGIDI into four major categories. Factors with a greater contribution are plotted closer to the centre, while those with a lesser contribution are plotted toward the edge. The information provided by these contributions can assist in the selection of appropriate parent contributors in crossbreeding programs. FA1 had a smaller effect on hybrids AVTOV1010×C41, AVTOV1001×C41 and AVTOV1002×C41, indicating that these hybrids were good performers for most FA1-related traits (), namely NFPC, NFrPC, FL, FW, AFW and NLPF. FA2 had the lowest effect on hybrids AVTOV1010×C41 and AVTOV1001×C41, indicating that these two hybrids have strengths in NFPP, YPP, Yield, DFPF and wilt. FA3 had a lower impact on the AVTOV1010×C41 hybrid, suggesting that this hybrid performed well for most of the FA3-correlated traits, namely the FSI, TSS, and TLCV. Finally, FA4 had a smaller effect on hybrids AVTOV1010×C41, AVTOV1001×C41 and AVTOV1002×C41, indicating that these three genotypes have strengths in PHLH and/or NPBLH. The ranking of selected genotypes based on their combinations of multiple traits has revealed that hybrids AVTOV1002×C41 and AVTOV1010×C41 are the two highest performing.

## 4 Experimental Design, Materials and Methods

### 4.1 Location and cultivation environment

The experiment was conducted at the Olericulture division of the Horticulture Research Centre (HRC) of Bangladesh Agricultural Research Institute (BARI), Gazipur-1701 (23°59′27.7″N 90°24′42.4″E, 8.4 masl). The climate of the experimental site is subtropical characterized by heavy rainfall from May to October and medium to scanty during the rest of the year. The monthly average minimum and maximum temperature during the crop period were 24.7 °C and 32.5 °C respectively. The monthly average relative humidity was 79.35 %. The monthly average rainfall during the crop period was 183.29 mm. The soil of the experimental site belongs to the general soil type (Shallow Red Brown). The top soils were clay loam in texture having soil pH ranging from 6.0- 6.6 and had organic matter of 0.84 %. The experimental area was flat having an available irrigation and drainage system and above flood level. The experimental area was enhanced with a recommended dose of fertilizers (550–450–250 kg/ha of urea, TSP, MOP and cow dung 10 t/ha).

### 4.2 Plant material and experimental design

The experiment was laid out in Randomized Complete Block Design (RCBD) with two replications. Seeds of selected 14 cross combinations from a Line × Tester mating design and their seven parental lines were the plant materials used for the study (seven female parents include AVTOV1001, AVTOV1002, AVTOV1006, AVTOV1007, AVOV1008, AVTOV1009 and AVTOV-1010 and two male genotypes were C41 and BARI-4 with diverse genetic bases and heat tolerance quality). Altogether, seeds of 23 genotypes were sown densely on 18th May 2012 in the primary seedbed. Forty-day-old tomato seedlings were transplanted in the main field under transparent polytunnels in the same location where F1 (experimental hybrids) were synthesized. The polytunnels were 2.3 m wide having two-unit beds with 0.8 *m* × 1 m sizes keeping a 30 cm drain in between 14-unit beds. Each unit bed contained double rows accommodating 24 plants.

### 4.3 Assessed traits and collection of data

The harvests began in the maturation stage and were carried out twice a week. Through the production cycle, five random competitive plants per treatment were selected, and observations were recorded. Observations for all the 16 characters described below were recorded for each of the genotypes and developed F1 hybrids (Table 4).

**Table 4:** Code, description and goal for selection of the traits evaluated.

SL No.	Code	Description	Unit	Goal
1	PHLH	Plant height at last harvest	cm	increase
2	NPBLH	Number of branches per plant	count	increase
3	NFPC	Number of flowers per cluster	count	increase
4	NFrPC	Number of fruits per cluster	count	increase
5	FL	Fruit length	cm	increase
6	FW	Fruit width	cm	increase
7	AFW	Average fruit weight	g	increase
8	NFPP	Number of fruits per plant	count	increase
9	YPP	Yield per plant	Kg	increase
10	FSI	Fruit shape index	ratio	increase
11	TSS	Total soluble solids	%	increase
12	Yield	Yield per hectare	Ton ha^−1^	increase
13	DFPF	Days to 50 per cent flowering	days	decrease
14	NLPF	Number of locules per fruit	count	decrease
15	TLCV	Number of infected plants in the plot ÷ Total number of plants in the plot	%	decrease
16	wilt	The disease incidence scale given by Mew and Ho [9]	%	decrease

### 4.4 Statistical analysis

The theory of the MGIDI index is arranged into four main steps to select the best genotypes based on statistics about multiple trait information [[6], [7], [8],^10^].

#### 4.4.1 Rescaling the traits

Let *X_ij_* be a two-way table with *i* rows/genotypes/treatments and *j* columns/traits. The rescaled value for the *i*th row and *j*th column (*rX_ij_*) is given by [6] :

(1) $$rX_{\text{ij}}=\frac{\left(\eta_{\text{nj}}-\varphi_{\text{nj}}\right)}{\left(\eta_{\text{oj}}-\varphi_{\text{oj}}\right)}x\left(\theta_{\text{ij}}-\eta_{\text{oj}}\right)+\eta_{\text{nj}}$$

Where *φ*_nj_ and *η*_oj_ represent the minimum and maximum original values for the *j*th trait, respectively, while *φ_nj_* and *η_nj_* represent the new minimum and maximum values for the *j*th trait after rescaling, respectively. The original value for the *j*th trait of the *i*th genotype is represented by θ*ij*. The values of *φ_nj_* and *η_nj_* were selected based on the desired gains for each trait: for traits with positive gains, *φ_nj_* = 0 and *η_nj_* = 100 was used, while for traits with negative gains, *φ_nj_* = 100 and *η_nj_* = 0 were used, as suggested by Olivoto and Nardino [7].

#### 4.4.2 Factor analysis

The second step is to compute an exploratory factor analysis (FA) with *rX_ij_* to account for the correlation structure and dimensionality reduction of the data, as follows:

(2) X=μ+Lf+ε

Where **X** is a *p* × 1 vector of rescaled observations; *µ* is a *p* × 1 vector of standardized means; **L** is a *p* *×* *f* matrix of factorial loadings; **f** is a *p* × 1 vector of common factors; and *ε* is a *p* × 1 vector of residuals, being *p* and *f*, the number of traits and common factors retained, respectively. The eigenvalues and eigenvectors are obtained from the correlation matrix of *rX_ij_*. The initial loadings are obtained considering only factors with eigenvalues higher than one. Then, the varimax rotation criteria [11] are used for the analytic rotation and estimation of final loadings. The scores are then obtained as follows:

(3) $$F=Z{\left(A^{T}R^{-1}\right)}^{T}$$

Where **F** is a *g* *×* *f* matrix with the factorial scores; **Z** is a *g* *×* *p* matrix with the (rescaled) standardized means; **A** is a *p* *×* *f* matrix of canonical loadings, and **R** is a *p* *×* *p* correlation matrix between the traits. *g, f* and *p* represent the number of rows/genotypes/treatments, and factors retained and analyzed traits, respectively.

#### 4.4.3 Ideotype planning

By definition [Eq. (1)], the ideotype has the maximum rescaled value (100) for all analyzed traits. Thus, the ideotype can be defined by a 1 × *p* vector I such that *I* = [100, 100, …….100]. The scores for I are also estimated according to Eq. (3).

#### 4.4.4 The MGIDI index

The fourth and last step is the estimation of the multi-trait genotype–ideotype distance index (MGIDI), which is used to rank the treatments based on the desired values of the studied trait, as follows [[6], [7], [8]]:

(4) $$\text{MGID}I_{i}={\left[\sum_{j=1}^{f}{\left(\gamma_{ij}-\gamma_{j}\right)}^{2}\right]}^{0.5}$$

Where *MGIDI_i_* is the multi-trait genotype–ideotype distance index for the *i*th row/genotype/treatment; *γij* is the score of the *i*th row/ genotype/treatment in the *j*th factor (*i* = 1, 2, …. *g; j* = 1, 2, …. *f*), being *g* and *f* the number of rows/genotypes/treatments and factors, respectively; and *γ_ij_* is the *j*th score of the ideotype. The row/genotype/treatment with the lowest MGIDI is then closer to the ideotype and therefore presents desired values for all the *p* traits. The selection differential for all traits was computed considering a selection intensity (10 %), i.e., the first two treatments/genotypes with the lowest MGIDI index were selected.

The proportion of the MGIDI index of the *i*th row/genotype/treatment explained by the *j*th factor (*ω_ij_*) is used to show the strengths and weaknesses of genotypes/treatments and is computed as [[6], [7], [8]]:

(5) $$\omega_{ij}=\frac{\sqrt{D_{ij}^{2}}}{\sum_{j=1}^{f}\sqrt{D_{ij}^{2}}}$$

Where *D_ij_* is the distance between the *i*th genotype/treatment and the ideotype for the *j*th factor. Low contributions of a factor indicate that the traits within such a factor are close to the ideotype.

Data manipulation and index calculation were performed in the R Software version 4.3.1 (R Core Team, 2024) using the package metan v1.18.0 [12].

## Limitations

None.

## Ethics Statement

All authors have read and followed the ethical requirements for publication in Data in Brief and our work meets these requirements. Our work does not involve studies with animals and humans.

## CRediT authorship contribution statement

**Mohammad Matin Akand:** Conceptualization, Methodology, Software, Validation, Formal analysis, Supervision, Investigation, Visualization, Writing – original draft, Writing – review & editing. **Mohammad Mostafa Kamal:** Data curation, Formal analysis, Writing – review & editing. **Md. Iqbal Haque:** Writing – review & editing. **Shyamal Brahma:** Writing – review & editing. **Mohammed Nure Yousuf:** Data curation, Writing – review & editing. **Mahmuda Khatun:** Data curation, Formal analysis.

## Declaration of competing interest

The authors declare that they have no known competing financial interests or personal relationships that could have appeared to influence the work reported in this paper.

## Data Availability

A dataset on multi-trait selection approach for the evaluation of F1 tomato hybrids along with their parents under hot and humid conditions in Bangladesh (Original data) (Mendeley Data).

## References

[1] Yunus M., Rahman M.S., Islam S., Sayadat N., Banik B., Islam A., Hassan M.F. (2023). Unveiling the factors influencing organic tomato (Solanum lycopersicum) production: an empirical evidence from the Mymensingh district of Bangladesh. South Asian J. Soc. Stud. Econ..

[2] Aldubai A.A., Alsadon A.A., Migdadi H.H., Alghamdi S.S., Al-Faifi S.A., Afzal M. (2022). Response of tomato (Solanum lycopersicum L.) genotypes to heat stress using morphological and expression study. Plants.

[3] Quamruzzaman A.K.M., Akter L., Islam F. (2023). Yield and pest performance of high-temperature tolerant tomato (Lycopersicon esculentum) lines for year-round tomato production in Bangladesh. Agric. Sci..

[4] Hajong P., Rahman M.H., Kobir M.S. (2022). Marketing system of summer tomato in Jashore district of Bangladesh. Int. J. Agric. Res. Innov. Technol..

[5] Kumar S., Singh V., Maurya P.K., Kumar B.A., Yadav P.K. (2017). Evaluation of F1 hybrids along with parents for yield and related characteristics in tomato (Solanum lycopersicum Child). Int. J. Curr. Microbiol. Appl. Sci..

[6] Olivoto T., Inês Diel M., Schmidt D., Dal’ A., Lúcio C. (2021). Multivariate analysis of strawberry experiments: where are we now and where can we go?. BioRxiv[Preprint].

[7] Olivoto T., Nardino M. (2021). MGIDI: toward an effective multivariate selection in biological experiments. Bioinformatics.

[8] Olivoto T., Diel M.I., Schmidt D., Lúcio A.D. (2022). MGIDI: a powerful tool to analyze plant multivariate data. Plant Methods.

[9] Mew T., Ho W. (1977). Effect of soil temperature on resistance of tomato cultivars to bacterial wilt. Phytopathology.

[10] Olivoto T., Lúcio A.D., da Silva J.A.G., Sari B.G., Diel M.I. (2019). Mean performance and stability in multi-environment trials II: selection based on multiple traits. Agron. J..

[11] Kaiser H.F. (1958). The varimax criterion for analytic rotation in factor analysis. Psychometrika.

[12] Olivoto T., Lúcio A.D. (2020). metan : an R package for multi-environment trial analysis. Methods Ecol. Evol..

